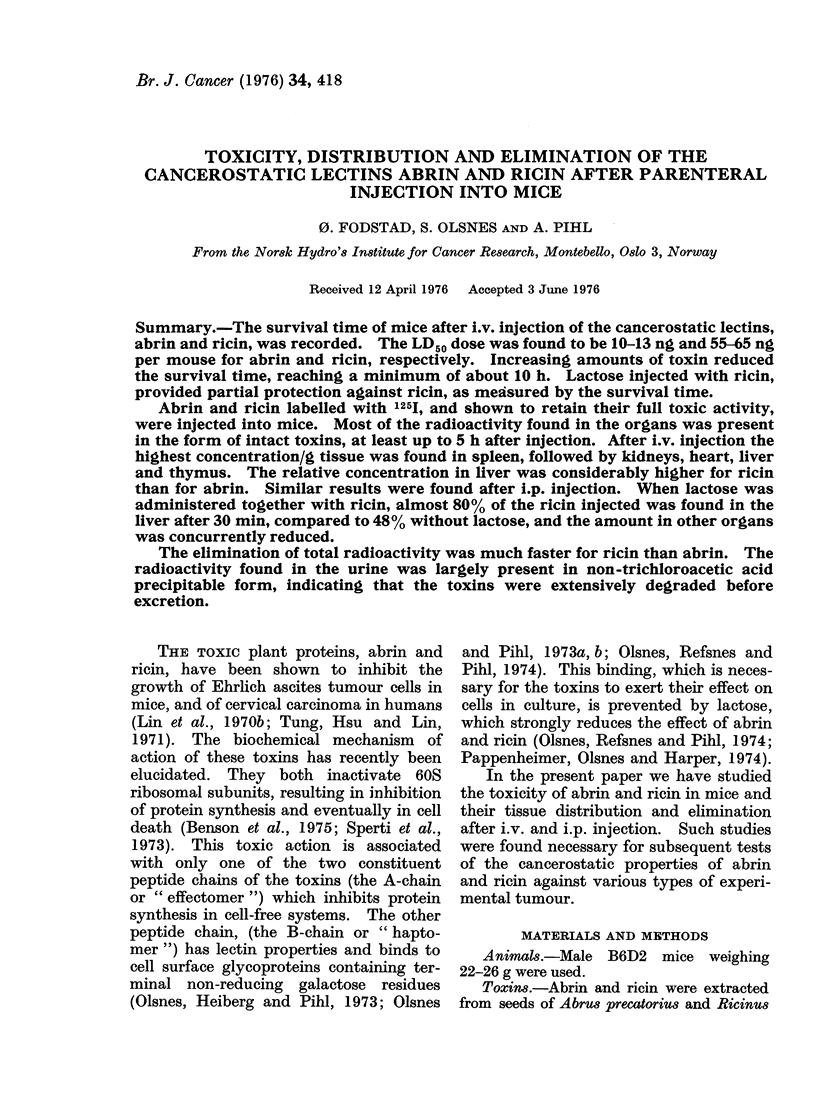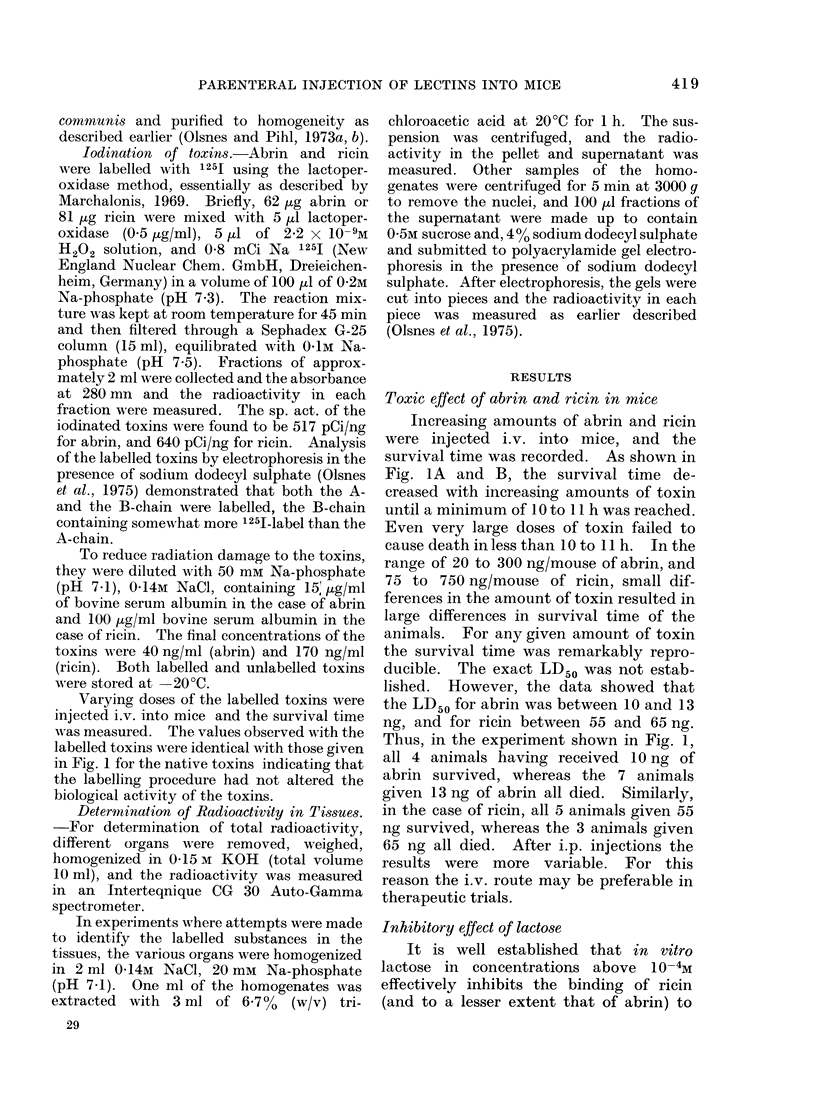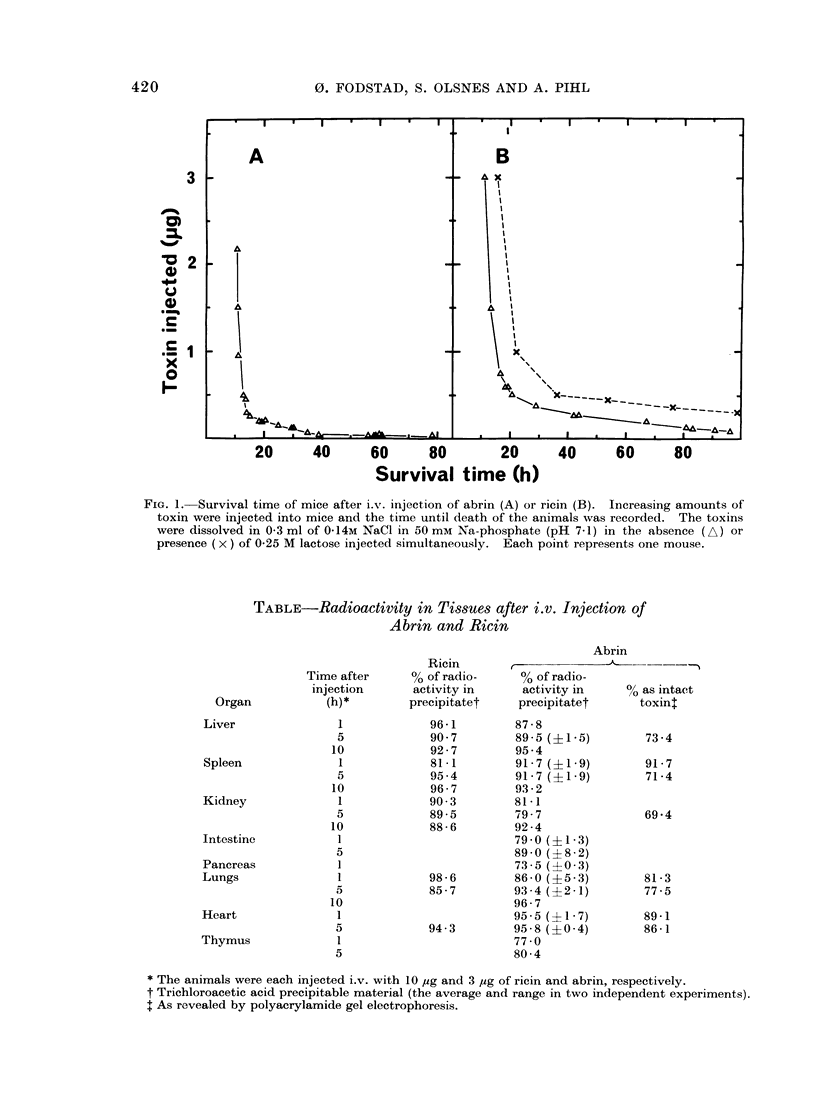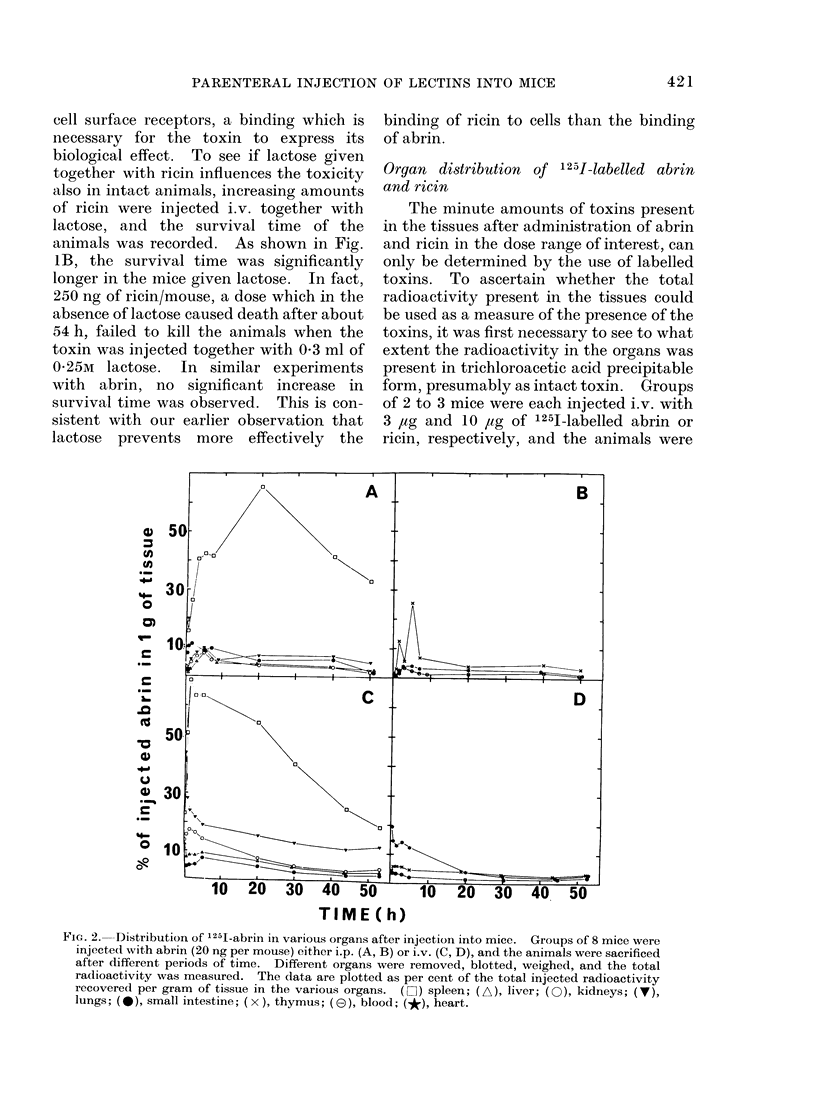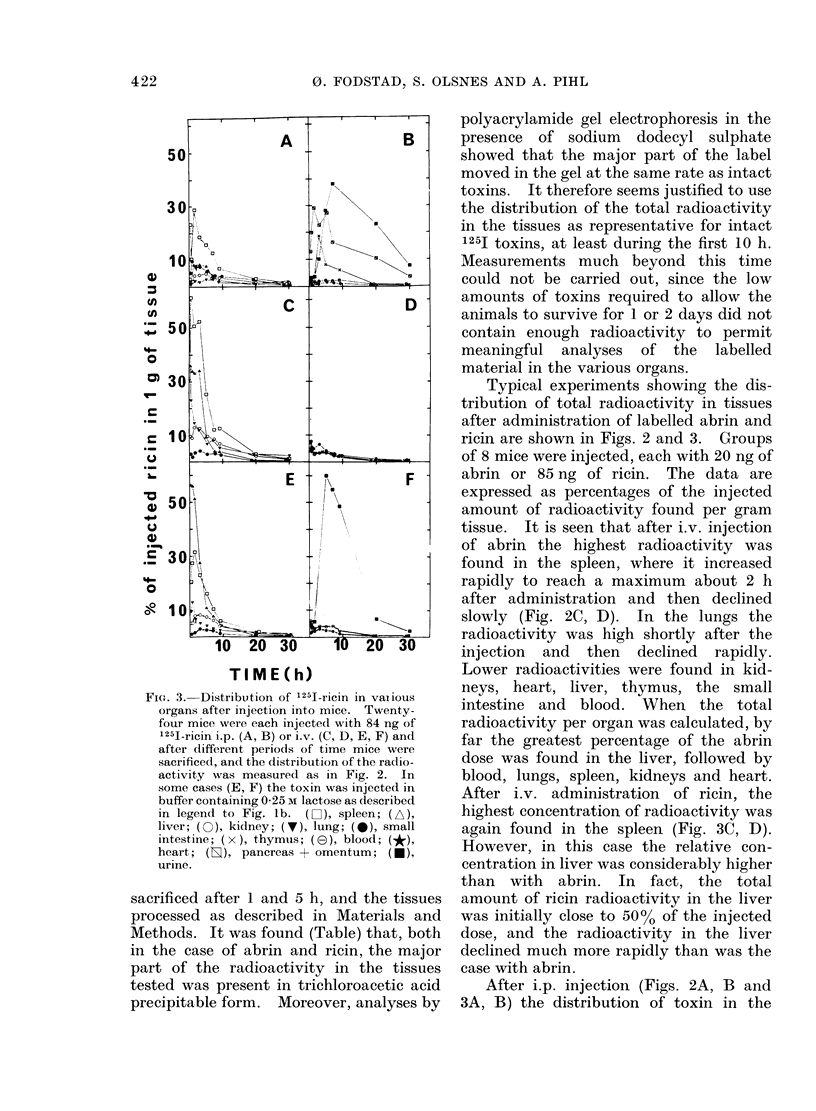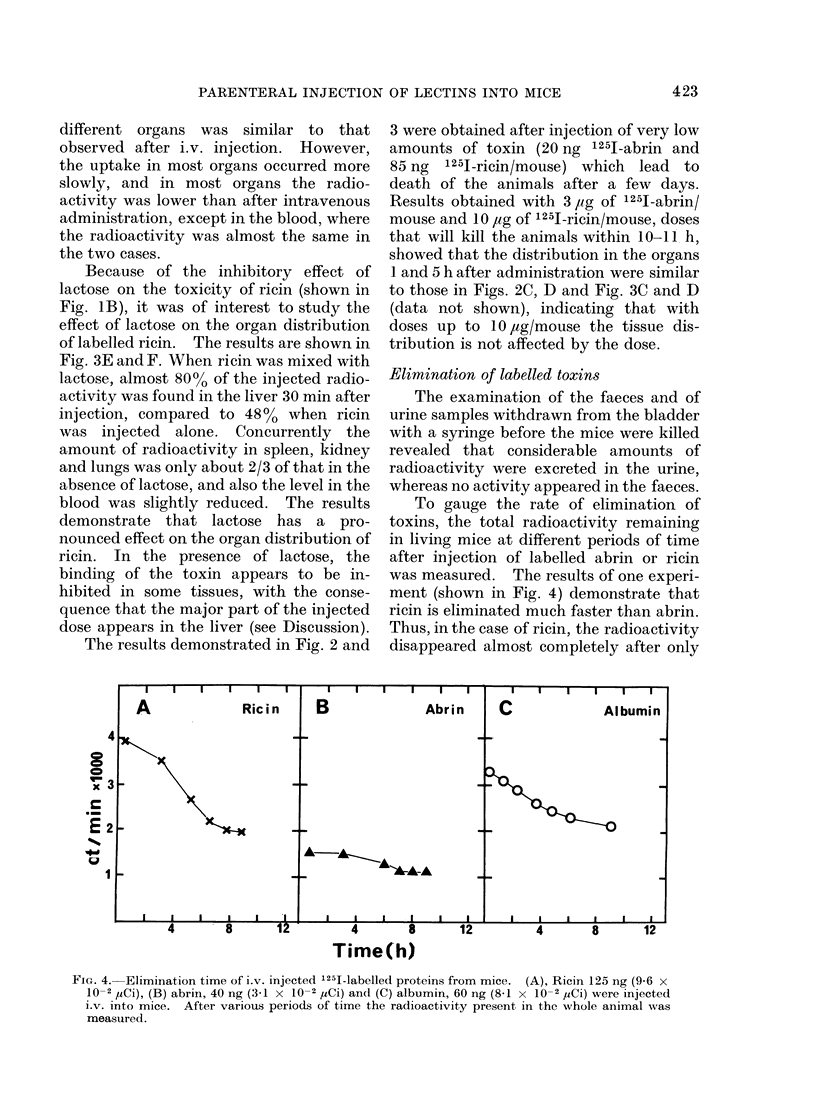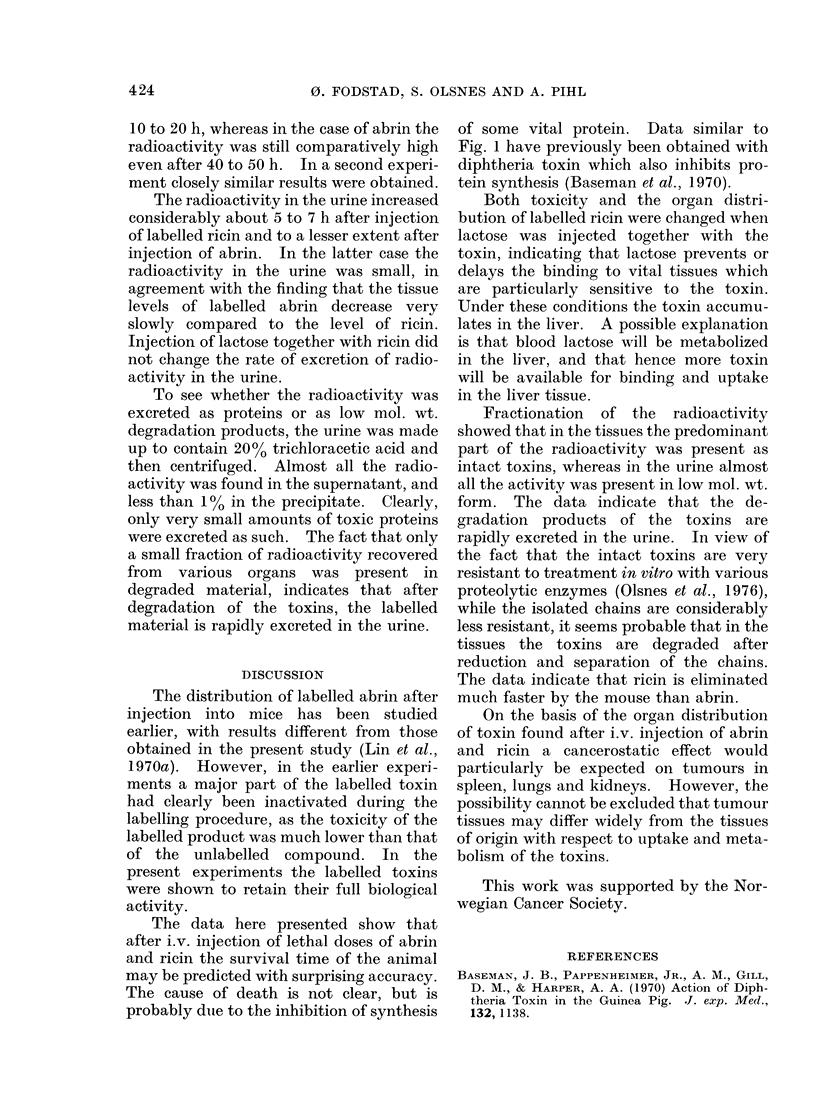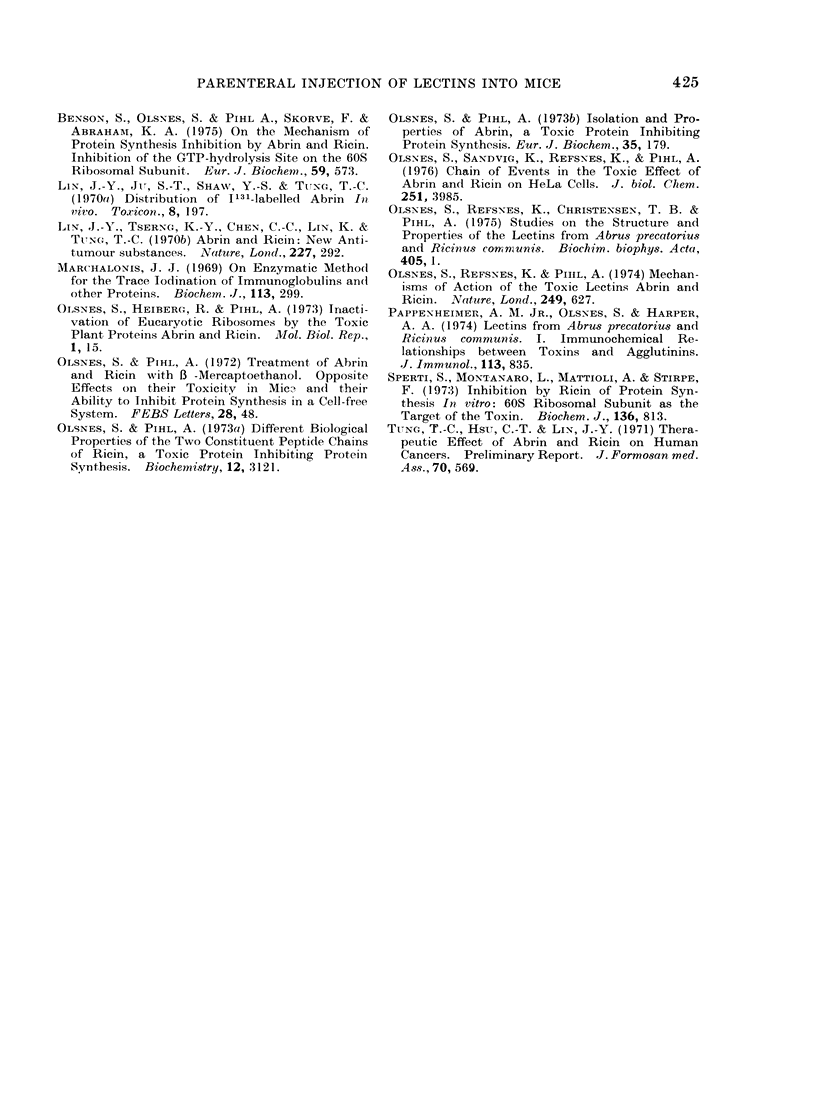# Toxicity, distribution and elimination of the cancerostatic lectins abrin and ricin after parenteral injection into mice.

**DOI:** 10.1038/bjc.1976.187

**Published:** 1976-10

**Authors:** O. Fodstad, S. Olsnes, A. Pihl

## Abstract

The survival time of mice after i.v. injection of the cancerostatic lectins, abrin and ricin was recorded. The LD50 dose was found to be 10-13 ng and 55-65 ng per mouse for abrin and ricin, respectively. Increasing amounts of toxin reduced the survival time, reaching a minimum of about 10 h. Lactose injected with ricin, provided partial protection against ricin, as measured by the survival time. Abrin and ricin labelled with 125I, and shown to retain their full toxic activity, were injected into mice. Most of the radioactivity found in the organs was present in the form of intact toxins, at least up to 5 h after injection. After i.v. injection the highest concentration/g tissue was found in spleen, followed by kidneys, heart, liver and thymus. The relative concentration in liver was considerably higher for ricin than for abrin. Similar results were found after i.p. injection. When lactose was administered together with ricin, almost 80% of the ricin injected was found in the liver after 30 min, compared to 48% without lactose, and the amount in other organs was concurrently reduced. The elimination of total radioactivity was much faster for ricin than abrin. The radioactivity found in the urine was largely present in non-trichloroacetic acid precipitable form, indicating that the toxins were extensively degraded before excretion.


*Br. J. Cancer* (1976) **34**, 418

### TOXICITY, DISTRIBUTION AND ELIMINATION OF THE CANCEROSTATIC LECTINS ABRIN AND RICIN AFTER PARENTERAL INJECTION INTO MICE

Ø. FODSTAD, S. OLSNES AND A. PIHL

*From the Norsk Hydro's Institute for Cancer Research, Montebello, Oslo 3, Norway*

Received 12 April 1976 Accepted 3 June 1976

**Summary.—The survival time of mice after i.v. injection of the cancerostatic lectins, abrin and ricin, was recorded. The $LD_{50}$ dose was found to be 10–13 ng and 55–65 ng per mouse for abrin and ricin, respectively. Increasing amounts of toxin reduced the survival time, reaching a minimum of about 10 h. Lactose injected with ricin, provided partial protection against ricin, as measured by the survival time.**

**Abrin and ricin labelled with $^{125}I$, and shown to retain their full toxic activity, were injected into mice. Most of the radioactivity found in the organs was present in the form of intact toxins, at least up to 5 h after injection. After i.v. injection the highest concentration/g tissue was found in spleen, followed by kidneys, heart, liver and thymus. The relative concentration in liver was considerably higher for ricin than for abrin. Similar results were found after i.p. injection. When lactose was administered together with ricin, almost 80% of the ricin injected was found in the liver after 30 min, compared to 48% without lactose, and the amount in other organs was concurrently reduced.**

**The elimination of total radioactivity was much faster for ricin than abrin. The radioactivity found in the urine was largely present in non-trichloroacetic acid precipitable form, indicating that the toxins were extensively degraded before excretion.**

THE TOXIC plant proteins, abrin and ricin, have been shown to inhibit the growth of Ehrlich ascites tumour cells in mice, and of cervical carcinoma in humans (Lin *et al.*, 1970*b*; Tung, Hsu and Lin, 1971). The biochemical mechanism of action of these toxins has recently been elucidated. They both inactivate 60S ribosomal subunits, resulting in inhibition of protein synthesis and eventually in cell death (Benson *et al.*, 1975; Sperti *et al.*, 1973). This toxic action is associated with only one of the two constituent peptide chains of the toxins (the A-chain or " effectomer ") which inhibits protein synthesis in cell-free systems. The other peptide chain, (the B-chain or " haptomer ") has lectin properties and binds to cell surface glycoproteins containing terminal non-reducing galactose residues (Olsnes, Heiberg and Pihl, 1973; Olsnes and Pihl, 1973*a*, *b*; Olsnes, Refsnes and Pihl, 1974). This binding, which is necessary for the toxins to exert their effect on cells in culture, is prevented by lactose, which strongly reduces the effect of abrin and ricin (Olsnes, Refsnes and Pihl, 1974; Pappenheimer, Olsnes and Harper, 1974).

In the present paper we have studied the toxicity of abrin and ricin in mice and their tissue distribution and elimination after i.v. and i.p. injection. Such studies were found necessary for subsequent tests of the cancerostatic properties of abrin and ricin against various types of experimental tumour.

### MATERIALS AND METHODS

*Animals.*—Male B6D2 mice weighing 22–26 g were used.

*Toxins.*—Abrin and ricin were extracted from seeds of *Abrus precatorius* and *Ricinus*

*communis* and purified to homogeneity as described earlier (Olsnes and Pihl, 1973*a*, *b*).

*Iodination of toxins.*—Abrin and ricin were labelled with $^{125}I$ using the lactoperoxidase method, essentially as described by Marchalonis, 1969. Briefly, 62 $\mu$g abrin or 81 $\mu$g ricin were mixed with 5 $\mu$l lactoperoxidase (0·5 $\mu$g/ml), 5 $\mu$l of $2{\cdot}2 \times 10^{-9}$M $H_2O_2$ solution, and 0·8 mCi Na $^{125}I$ (New England Nuclear Chem. GmbH, Dreieichenheim, Germany) in a volume of 100 $\mu$l of 0·2M Na-phosphate (pH 7·3). The reaction mixture was kept at room temperature for 45 min and then filtered through a Sephadex G-25 column (15 ml), equilibrated with 0·1M Na-phosphate (pH 7·5). Fractions of approxmately 2 ml were collected and the absorbance at 280 mn and the radioactivity in each fraction were measured. The sp. act. of the iodinated toxins were found to be 517 pCi/ng for abrin, and 640 pCi/ng for ricin. Analysis of the labelled toxins by electrophoresis in the presence of sodium dodecyl sulphate (Olsnes *et al.*, 1975) demonstrated that both the A- and the B-chain were labelled, the B-chain containing somewhat more $^{125}I$-label than the A-chain.

To reduce radiation damage to the toxins, they were diluted with 50 mM Na-phosphate (pH 7·1), 0·14M NaCl, containing 15 $\mu$g/ml of bovine serum albumin in the case of abrin and 100 $\mu$g/ml bovine serum albumin in the case of ricin. The final concentrations of the toxins were 40 ng/ml (abrin) and 170 ng/ml (ricin). Both labelled and unlabelled toxins were stored at −20°C.

Varying doses of the labelled toxins were injected i.v. into mice and the survival time was measured. The values observed with the labelled toxins were identical with those given in Fig. 1 for the native toxins indicating that the labelling procedure had not altered the biological activity of the toxins.

*Determination of Radioactivity in Tissues.*—For determination of total radioactivity, different organs were removed, weighed, homogenized in 0·15 M KOH (total volume 10 ml), and the radioactivity was measured in an Interteqnique CG 30 Auto-Gamma spectrometer.

In experiments where attempts were made to identify the labelled substances in the tissues, the various organs were homogenized in 2 ml 0·14M NaCl, 20 mM Na-phosphate (pH 7·1). One ml of the homogenates was extracted with 3 ml of 6·7% (w/v) trichloroacetic acid at 20°C for 1 h. The suspension was centrifuged, and the radioactivity in the pellet and supernatant was measured. Other samples of the homogenates were centrifuged for 5 min at 3000 *g* to remove the nuclei, and 100 $\mu$l fractions of the supernatant were made up to contain 0·5M sucrose and, 4% sodium dodecyl sulphate and submitted to polyacrylamide gel electrophoresis in the presence of sodium dodecyl sulphate. After electrophoresis, the gels were cut into pieces and the radioactivity in each piece was measured as earlier described (Olsnes *et al.*, 1975).

### RESULTS

### *Toxic effect of abrin and ricin in mice*

Increasing amounts of abrin and ricin were injected i.v. into mice, and the survival time was recorded. As shown in Fig. 1A and B, the survival time decreased with increasing amounts of toxin until a minimum of 10 to 11 h was reached. Even very large doses of toxin failed to cause death in less than 10 to 11 h. In the range of 20 to 300 ng/mouse of abrin, and 75 to 750 ng/mouse of ricin, small differences in the amount of toxin resulted in large differences in survival time of the animals. For any given amount of toxin the survival time was remarkably reproducible. The exact $LD_{50}$ was not established. However, the data showed that the $LD_{50}$ for abrin was between 10 and 13 ng, and for ricin between 55 and 65 ng. Thus, in the experiment shown in Fig. 1, all 4 animals having received 10 ng of abrin survived, whereas the 7 animals given 13 ng of abrin all died. Similarly, in the case of ricin, all 5 animals given 55 ng survived, whereas the 3 animals given 65 ng all died. After i.p. injections the results were more variable. For this reason the i.v. route may be preferable in therapeutic trials.

### *Inhibitory effect of lactose*

It is well established that *in vitro* lactose in concentrations above $10^{-4}$M effectively inhibits the binding of ricin (and to a lesser extent that of abrin) to

FIG. 1.—Survival time of mice after i.v. injection of abrin (A) or ricin (B). Increasing amounts of toxin were injected into mice and the time until death of the animals was recorded. The toxins were dissolved in 0·3 ml of 0·14M NaCl in 50 mM Na-phosphate (pH 7·1) in the absence (△) or presence (×) of 0·25 M lactose injected simultaneously. Each point represents one mouse.

TABLE—*Radioactivity in Tissues after i.v. Injection of Abrin and Ricin*

| Organ | Time after injection (h)* | Ricin % of radio-activity in precipitate† | Abrin % of radio-activity in precipitate† | Abrin % as intact toxin‡ |
|---|---|---|---|---|
| Liver | 1 | 96·1 | 87·8 | |
| | 5 | 90·7 | 89·5 (±1·5) | 73·4 |
| | 10 | 92·7 | 95·4 | |
| Spleen | 1 | 81·1 | 91·7 (±1·9) | 91·7 |
| | 5 | 95·4 | 91·7 (±1·9) | 71·4 |
| | 10 | 96·7 | 93·2 | |
| Kidney | 1 | 90·3 | 81·1 | |
| | 5 | 89·5 | 79·7 | 69·4 |
| | 10 | 88·6 | 92·4 | |
| Intestine | 1 | | 79·0 (±1·3) | |
| | 5 | | 89·0 (±8·2) | |
| Pancreas | 1 | | 73·5 (±0·3) | |
| Lungs | 1 | 98·6 | 86·0 (±5·3) | 81·3 |
| | 5 | 85·7 | 93·4 (±2·1) | 77·5 |
| | 10 | | 96·7 | |
| Heart | 1 | | 95·5 (±1·7) | 89·1 |
| | 5 | 94·3 | 95·8 (±0·4) | 86·1 |
| Thymus | 1 | | 77·0 | |
| | 5 | | 80·4 | |

* The animals were each injected i.v. with 10 μg and 3 μg of ricin and abrin, respectively.
† Trichloroacetic acid precipitable material (the average and range in two independent experiments).
‡ As revealed by polyacrylamide gel electrophoresis.

cell surface receptors, a binding which is necessary for the toxin to express its biological effect. To see if lactose given together with ricin influences the toxicity also in intact animals, increasing amounts of ricin were injected i.v. together with lactose, and the survival time of the animals was recorded. As shown in Fig. 1B, the survival time was significantly longer in the mice given lactose. In fact, 250 ng of ricin/mouse, a dose which in the absence of lactose caused death after about 54 h, failed to kill the animals when the toxin was injected together with 0·3 ml of 0·25M lactose. In similar experiments with abrin, no significant increase in survival time was observed. This is consistent with our earlier observation that lactose prevents more effectively the binding of ricin to cells than the binding of abrin.

*Organ distribution of* $^{125}$*I-labelled abrin and ricin*

The minute amounts of toxins present in the tissues after administration of abrin and ricin in the dose range of interest, can only be determined by the use of labelled toxins. To ascertain whether the total radioactivity present in the tissues could be used as a measure of the presence of the toxins, it was first necessary to see to what extent the radioactivity in the organs was present in trichloroacetic acid precipitable form, presumably as intact toxin. Groups of 2 to 3 mice were each injected i.v. with 3 $\mu$g and 10 $\mu$g of $^{125}$I-labelled abrin or ricin, respectively, and the animals were

FIG. 2.—Distribution of $^{125}$I-abrin in various organs after injection into mice. Groups of 8 mice were injected with abrin (20 ng per mouse) either i.p. (A, B) or i.v. (C, D), and the animals were sacrificed after different periods of time. Different organs were removed, blotted, weighed, and the total radioactivity was measured. The data are plotted as per cent of the total injected radioactivity recovered per gram of tissue in the various organs. (□) spleen; (△), liver; (○), kidneys; (▼), lungs; (●), small intestine; (×), thymus; (⊖), blood; (★), heart.

FIG. 3.—Distribution of $^{125}$I-ricin in various organs after injection into mice. Twenty-four mice were each injected with 84 ng of $^{125}$I-ricin i.p. (A, B) or i.v. (C, D, E, F) and after different periods of time mice were sacrificed, and the distribution of the radioactivity was measured as in Fig. 2. In some cases (E, F) the toxin was injected in buffer containing 0·25 M lactose as described in legend to Fig. 1b. (□), spleen; (△), liver; (○), kidney; (▼), lung; (●), small intestine; (×), thymus; (⊖), blood; (★), heart; (⧅), pancreas + omentum; (■), urine.

sacrificed after 1 and 5 h, and the tissues processed as described in Materials and Methods. It was found (Table) that, both in the case of abrin and ricin, the major part of the radioactivity in the tissues tested was present in trichloroacetic acid precipitable form. Moreover, analyses by polyacrylamide gel electrophoresis in the presence of sodium dodecyl sulphate showed that the major part of the label moved in the gel at the same rate as intact toxins. It therefore seems justified to use the distribution of the total radioactivity in the tissues as representative for intact $^{125}$I toxins, at least during the first 10 h. Measurements much beyond this time could not be carried out, since the low amounts of toxins required to allow the animals to survive for 1 or 2 days did not contain enough radioactivity to permit meaningful analyses of the labelled material in the various organs.

Typical experiments showing the distribution of total radioactivity in tissues after administration of labelled abrin and ricin are shown in Figs. 2 and 3. Groups of 8 mice were injected, each with 20 ng of abrin or 85 ng of ricin. The data are expressed as percentages of the injected amount of radioactivity found per gram tissue. It is seen that after i.v. injection of abrin the highest radioactivity was found in the spleen, where it increased rapidly to reach a maximum about 2 h after administration and then declined slowly (Fig. 2C, D). In the lungs the radioactivity was high shortly after the injection and then declined rapidly. Lower radioactivities were found in kidneys, heart, liver, thymus, the small intestine and blood. When the total radioactivity per organ was calculated, by far the greatest percentage of the abrin dose was found in the liver, followed by blood, lungs, spleen, kidneys and heart. After i.v. administration of ricin, the highest concentration of radioactivity was again found in the spleen (Fig. 3C, D). However, in this case the relative concentration in liver was considerably higher than with abrin. In fact, the total amount of ricin radioactivity in the liver was initially close to 50% of the injected dose, and the radioactivity in the liver declined much more rapidly than was the case with abrin.

After i.p. injection (Figs. 2A, B and 3A, B) the distribution of toxin in the different organs was similar to that observed after i.v. injection. However, the uptake in most organs occurred more slowly, and in most organs the radioactivity was lower than after intravenous administration, except in the blood, where the radioactivity was almost the same in the two cases.

Because of the inhibitory effect of lactose on the toxicity of ricin (shown in Fig. 1B), it was of interest to study the effect of lactose on the organ distribution of labelled ricin. The results are shown in Fig. 3E and F. When ricin was mixed with lactose, almost 80% of the injected radioactivity was found in the liver 30 min after injection, compared to 48% when ricin was injected alone. Concurrently the amount of radioactivity in spleen, kidney and lungs was only about 2/3 of that in the absence of lactose, and also the level in the blood was slightly reduced. The results demonstrate that lactose has a pronounced effect on the organ distribution of ricin. In the presence of lactose, the binding of the toxin appears to be inhibited in some tissues, with the consequence that the major part of the injected dose appears in the liver (see Discussion).

The results demonstrated in Fig. 2 and 3 were obtained after injection of very low amounts of toxin (20 ng $^{125}$I-abrin and 85 ng $^{125}$I-ricin/mouse) which lead to death of the animals after a few days. Results obtained with 3 $\mu$g of $^{125}$I-abrin/mouse and 10 $\mu$g of $^{125}$I-ricin/mouse, doses that will kill the animals within 10–11 h, showed that the distribution in the organs 1 and 5 h after administration were similar to those in Figs. 2C, D and Fig. 3C and D (data not shown), indicating that with doses up to 10 $\mu$g/mouse the tissue distribution is not affected by the dose.

*Elimination of labelled toxins*

The examination of the faeces and of urine samples withdrawn from the bladder with a syringe before the mice were killed revealed that considerable amounts of radioactivity were excreted in the urine, whereas no activity appeared in the faeces.

To gauge the rate of elimination of toxins, the total radioactivity remaining in living mice at different periods of time after injection of labelled abrin or ricin was measured. The results of one experiment (shown in Fig. 4) demonstrate that ricin is eliminated much faster than abrin. Thus, in the case of ricin, the radioactivity disappeared almost completely after only

FIG. 4.—Elimination time of i.v. injected $^{125}$I-labelled proteins from mice. (A), Ricin 125 ng (9·6 × $10^{-2}$ $\mu$Ci), (B) abrin, 40 ng (3·1 × $10^{-2}$ $\mu$Ci) and (C) albumin, 60 ng (8·1 × $10^{-2}$ $\mu$Ci) were injected i.v. into mice. After various periods of time the radioactivity present in the whole animal was measured.

10 to 20 h, whereas in the case of abrin the radioactivity was still comparatively high even after 40 to 50 h. In a second experiment closely similar results were obtained.

The radioactivity in the urine increased considerably about 5 to 7 h after injection of labelled ricin and to a lesser extent after injection of abrin. In the latter case the radioactivity in the urine was small, in agreement with the finding that the tissue levels of labelled abrin decrease very slowly compared to the level of ricin. Injection of lactose together with ricin did not change the rate of excretion of radioactivity in the urine.

To see whether the radioactivity was excreted as proteins or as low mol. wt. degradation products, the urine was made up to contain 20% trichloracetic acid and then centrifuged. Almost all the radioactivity was found in the supernatant, and less than 1% in the precipitate. Clearly, only very small amounts of toxic proteins were excreted as such. The fact that only a small fraction of radioactivity recovered from various organs was present in degraded material, indicates that after degradation of the toxins, the labelled material is rapidly excreted in the urine.

### DISCUSSION

The distribution of labelled abrin after injection into mice has been studied earlier, with results different from those obtained in the present study (Lin *et al.*, 1970*a*). However, in the earlier experiments a major part of the labelled toxin had clearly been inactivated during the labelling procedure, as the toxicity of the labelled product was much lower than that of the unlabelled compound. In the present experiments the labelled toxins were shown to retain their full biological activity.

The data here presented show that after i.v. injection of lethal doses of abrin and ricin the survival time of the animal may be predicted with surprising accuracy. The cause of death is not clear, but is probably due to the inhibition of synthesis of some vital protein. Data similar to Fig. 1 have previously been obtained with diphtheria toxin which also inhibits protein synthesis (Baseman *et al.*, 1970).

Both toxicity and the organ distribution of labelled ricin were changed when lactose was injected together with the toxin, indicating that lactose prevents or delays the binding to vital tissues which are particularly sensitive to the toxin. Under these conditions the toxin accumulates in the liver. A possible explanation is that blood lactose will be metabolized in the liver, and that hence more toxin will be available for binding and uptake in the liver tissue.

Fractionation of the radioactivity showed that in the tissues the predominant part of the radioactivity was present as intact toxins, whereas in the urine almost all the activity was present in low mol. wt. form. The data indicate that the degradation products of the toxins are rapidly excreted in the urine. In view of the fact that the intact toxins are very resistant to treatment *in vitro* with various proteolytic enzymes (Olsnes *et al.*, 1976), while the isolated chains are considerably less resistant, it seems probable that in the tissues the toxins are degraded after reduction and separation of the chains. The data indicate that ricin is eliminated much faster by the mouse than abrin.

On the basis of the organ distribution of toxin found after i.v. injection of abrin and ricin a cancerostatic effect would particularly be expected on tumours in spleen, lungs and kidneys. However, the possibility cannot be excluded that tumour tissues may differ widely from the tissues of origin with respect to uptake and metabolism of the toxins.

This work was supported by the Norwegian Cancer Society.

### REFERENCES

Baseman, J. B., Pappenheimer, Jr., A. M., Gill, D. M., & Harper, A. A. (1970) Action of Diphtheria Toxin in the Guinea Pig. *J. exp. Med.*, **132,** 1138.

Benson, S., Olsnes, S. & Pihl A., Skorve, F. & Abraham, K. A. (1975) On the Mechanism of Protein Synthesis Inhibition by Abrin and Ricin. Inhibition of the GTP-hydrolysis Site on the 60S Ribosomal Subunit. *Eur. J. Biochem.*, **59**, 573.

Lin, J.-Y., Ju, S.-T., Shaw, Y.-S. & Tung, T.-C. (1970*a*) Distribution of $I^{131}$-labelled Abrin *In vivo*. *Toxicon.*, **8**, 197.

Lin, J.-Y., Tserng, K.-Y., Chen, C.-C., Lin, K. & Tung, T.-C. (1970*b*) Abrin and Ricin: New Anti-tumour substances. *Nature, Lond.*, **227**, 292.

Marchalonis, J. J. (1969) On Enzymatic Method for the Trace Iodination of Immunoglobulins and other Proteins. *Biochem. J.*, **113**, 299.

Olsnes, S., Heiberg, R. & Pihl, A. (1973) Inactivation of Eucaryotic Ribosomes by the Toxic Plant Proteins Abrin and Ricin. *Mol. Biol. Rep.*, **1**, 15.

Olsnes, S. & Pihl, A. (1972) Treatment of Abrin and Ricin with ß -Mercaptoethanol. Opposite Effects on their Toxicity in Mice and their Ability to Inhibit Protein Synthesis in a Cell-free System. *FEBS Letters*, **28**, 48.

Olsnes, S. & Pihl, A. (1973*a*) Different Biological Properties of the Two Constituent Peptide Chains of Ricin, a Toxic Protein Inhibiting Protein Synthesis. *Biochemistry*, **12**, 3121.

Olsnes, S. & Pihl, A. (1973*b*) Isolation and Properties of Abrin, a Toxic Protein Inhibiting Protein Synthesis. *Eur. J. Biochem.*, **35**, 179.

Olsnes, S., Sandvig, K., Refsnes, K., & Pihl, A. (1976) Chain of Events in the Toxic Effect of Abrin and Ricin on HeLa Cells. *J. biol. Chem.* **251**, 3985.

Olsnes, S., Refsnes, K., Christensen, T. B. & Pihl, A. (1975) Studies on the Structure and Properties of the Lectins from *Abrus precatorius* and *Ricinus communis*. *Biochim. biophys. Acta*, **405**, 1.

Olsnes, S., Refsnes, K. & Pihl, A. (1974) Mechanisms of Action of the Toxic Lectins Abrin and Ricin. *Nature, Lond.*, **249**, 627.

Pappenheimer, A. M. Jr., Olsnes, S. & Harper, A. A. (1974) Lectins from *Abrus precatorius* and *Ricinus communis*. I. Immunochemical Relationships between Toxins and Agglutinins. *J. Immunol.*, **113**, 835.

Sperti, S., Montanaro, L., Mattioli, A. & Stirpe, F. (1973) Inhibition by Ricin of Protein Synthesis *In vitro*: 60S Ribosomal Subunit as the Target of the Toxin. *Biochem. J.*, **136**, 813.

Tung, T.-C., Hsu, C.-T. & Lin, J.-Y. (1971) Therapeutic Effect of Abrin and Ricin on Human Cancers. Preliminary Report. *J. Formosan med. Ass.*, **70**, 569.